# Turkish Validity and Reliability Study of Humanistic Practice Ability of Nursing Scale

**DOI:** 10.1155/2022/8435530

**Published:** 2022-01-31

**Authors:** Safiye Yanmış, Gülcan Bahçecioğlu Turan, Zülfünaz Özer

**Affiliations:** ^1^Department of Nursing, Faculty of Health Sciences, Erzican Binali Yıldırım University, Erzincan, Turkey; ^2^Department of Nursing, Faculty of Health Sciences, Fırat University, Elaziğ, Turkey; ^3^Istanbul Sabahattin Zaim University, Faculty of Health Sciences, Istanbul, Turkey

## Abstract

**Background:**

Nurses should have humanistic qualities to identify what the patient needs with an effective care plan and meet these needs. The aim of this study is to conduct the Turkish validity and reliability study of the Humanistic Practice Ability of Nursing Scale.

**Methods:**

This methodological study was carried out with 300 nurses online (e-mail, WhatsApp, Facebook, and Instagram) between April 02, 2021, and May 15, 2021. A questionnaire prepared via docs.google.com/forms was sent to the nurses. The scale was translated into Turkish, and then, the content and construct validity of its Turkish version was obtained through exploratory factor analysis and confirmatory factor analysis. The reliability of the scale was tested by performing item analysis and internal consistency analysis.

**Results:**

The analysis revealed that the content validity index of the scale was 0.96. In the exploratory factor analysis performed within the scope of the Turkish adaptation of the scale, the rate of the total variance explained was 59.63%. Factor loadings of all the items ranged between 0.47 and 0.79. While Cronbach's Alpha value was 0.93 for the overall Humanistic Practice Ability of Nursing Scale, it ranged from 0.71 to 0.89 for its subscales. The exploratory factor analysis and confirmatory factor analysis revealed no necessity of making any revision or modification in the scale. Therefore, the 29-item and five-dimension structure of the Humanistic Practice Ability of Nursing Scale was used in its Turkish version. Goodness-of-fit index values were obtained after the CFA was performed.

**Conclusion:**

The Turkish version of the Humanistic Practice Ability of Nursing Scale can be used as a valid and reliable measurement tool to assess nurses' humanistic practice ability.

## 1. Introduction

Human beings need care provided by others in every period of their life, starting from birth. The recovery of a sick individual is based on healthcare, which is the core or even the essence of nursing [1, 2]. Healthcare practices are tailored to the individuals' feelings, thoughts, and needs, by giving them sincere love and compassion and providing support [3]. Applying a scientific, ethical, and humanistic care to patients greatly contributes to patient health [1]. Nursing is defined as a profession in which human relations are actively experienced and humanistic philosophy is at the forefront [4]. Connect humanism (existence) has been explained as a helpful, beneficial, and positive phenomenon [5]. Human being defined as a unique being above all else according to the humanistic view needs help in both health and illness since its existence [6]. Nursing is one of the professions that take an active role in providing such help [7]. Humanistic nursing means more than a one-way, benevolent, nurse-patient relationship in which a purposeful call and response exists [8]. Being a must for social development and balanced nurse-patient relationships, humanistic nursing is considered as an internal requirement of nursing science [9, 10]. A nurse has the responsibility to know how the individuals to whom he/she gives care experience their own existence and to recognize and develop his/her own existence [11]. The humanistic values include the concepts such as existence, humanism, getting to know, understanding, empathizing and caring people, and individualistic approach. [12].

It is important for nurses to observe the values of humanism because the essence and focus of nursing is care [9, 10]. In addition, nurses should have humanistic qualities to identify what the patient needs with an effective care plan and meet these needs [6]. However, nurses often overlook humanistic features necessary to provide quality care [13], which sometimes causes conflicts in the relationships between nurses and patients. Currently, the nursing profession attaches great importance to the development of humanistic practice ability in the delivery of care [14]. Thanks to the investigation of the humanistic practice ability in nursing practices, improvement of care-related results as well as the factors required for nurses to improve their humanistic practice ability may be accurately analyzed. Presence of humanistic practice ability ensures more realistic, human-centered nursing, promotes patient health, reduces medical expenses, and improves patient satisfaction and prognosis [15–17]. One of the effective ways used in enhancing the quality of care provided by nurses is humanistic practice ability [17]. The introduction of humanistic practice ability is very important in the development of the spirit of humanistic nursing, humanistic clinical practices, and humanistic nursing education [18]. Humanistic care has the state of rationality that makes it difficult to embody objectively in practice. However, humanistic practice ability should be assessed with a scientific and standardized scale [19]. The literature review about the presence of a scale used to measure humanistic practice ability demonstrated the lack of such a scale in Turkey. Accordingly, the present study aims to adapt the Humanistic Practice Ability of Nursing Scale into Turkish and to test whether or not its Turkish version is a valid and reliable measurement instrument.

## 2. Materials and Methods

### 2.1. Design and Sample

This study was conducted in a methodological design. Nurses who were over the age of 18 years, were able to read and write in Turkish, were currently working in the profession, and were contacted through an online platform (e-mail, WhatsApp, Facebook, and Instagram) between April 02, 2021, and May 15, 2021, were included in the study. A questionnaire prepared via docs.google.com/forms was sent to the nurses via the abovementioned online tools. In the literature, the sample size is recommended to be at least fivefold to tenfold the number of the items in the scale for validity and reliability studies [20]. Therefore, the present study aimed to reach at least 290 people, which was 10 times the number of the items (*n* = 29) in the HPANS. Considering the possibility of losses during the study, 10 more people were included. Thus, the study was completed with 300 nurses who met the inclusion criteria. 30 nurses were included in the sample for test-retest analysis. Then, 30 nurses who underwent retesting were excluded from the study.

### 2.2. Data Collection Tools

The researchers collected the data online by using “Personal Information Form” and “Humanistic Practice Ability of Nursing Scale (HPANS).”

#### 2.2.1. Personal Information Form

This form was prepared by the researchers. It has six questions about the sociodemographic characteristics of the participants such as age, gender, marital status, education level, the unit they work in, and the duration of their working in the profession.

#### 2.2.2. Humanistic Practice Ability of Nursing Scale (HPANS)

The scale developed by Zhang et al. [19] in 2021 has 29 items and 5 subscales: nursing communication ability (items 1–7), psychological adjustment ability (items 8–11), ethics and legal application ability (items 12–18), nursing esthetic ability (items 19–21), and caring practical ability (items 22–29). Items of this five-point Likert-type scale are rated between 1 and 5 points (1 point: strongly disagree, 2 points: disagree, 3 points: undecided, 4 points: agree, 5 points: strongly agree). The sum of the scores of the 29 items yields the total HPANS score. The minimum and maximum scores of the scale are 29 and 145, respectively. High scores signify that nurses have a high level of humanistic practice ability. While the Cronbach's alpha (*α*) coefficient of the overall HPANS was 0.96, it ranged between 0.87 and 0.98 for its subscales [19].

*(1) Language Validity*. When a scale is translated into another language, it may become necessary to make changes in the existing structure of the original version of the scale due to conceptualization and expression differences. In order to make minimum changes, the items in the scale should be carefully reviewed. In order to make them meaningful in the translated language, they should be appropriately adapted and should be standardized according to the norms of the individuals using the translated language. If this process is not meticulously performed, the psychometric (reliability and validity) results of the scale may be unsatisfactory. Therefore, in the translation process, translators should be carefully selected and the appropriate translation technique should be implemented [21]. Therefore, the HPANS was translated into Turkish by two nursing academicians and an English linguist academic who was proficient in both English and Turkish. Later, this form was back translated by an English linguist who was familiar with the English culture and had a good command of English. This back translation was compared with the original version of the scale and its Turkish version was put into final form with the best translations of the original version. The translations in the Turkish version that best express each item in the original English version of the scale were selected and presented to five experts to obtain their views and suggestions. Then, the researchers reviewed the text by taking into account the experts' suggestions, and the Turkish version of the scale was put into final form and made ready for the validity and reliability studies.

*(2) Content Validity*. The researchers sent the instructions explaining the scale and a file including the English and Turkish version of the scale to experts via e-mail in order to evaluate them. It is reported in the literature that opinions from minimum 3 and maximum 20 experts are needed to calculate content validity index (CVI) [22]. In the present study, the researchers calculated CVI based on the opinions of 5 experts in line with the literature. CVI was determined by the Davis technique. According to the Davis technique, expert opinions are rated as follows: (4) “Totally Appropriate,” (3) “Very Appropriate,” (2) “Appropriate,” and (1) “Not Appropriate.” In this technique, the CVI value should be ≥ 0.80, which means that the item is sufficient in terms of content validity [23]. Based on the 5 faculty members' evaluation about the scale items for the content validity, the CVI value was determined as 0.96. As this calculated value was higher than the accepted criterion value, it was decided that the items were acceptable. Therefore, no item was removed from the scale.

**Figure 1:**
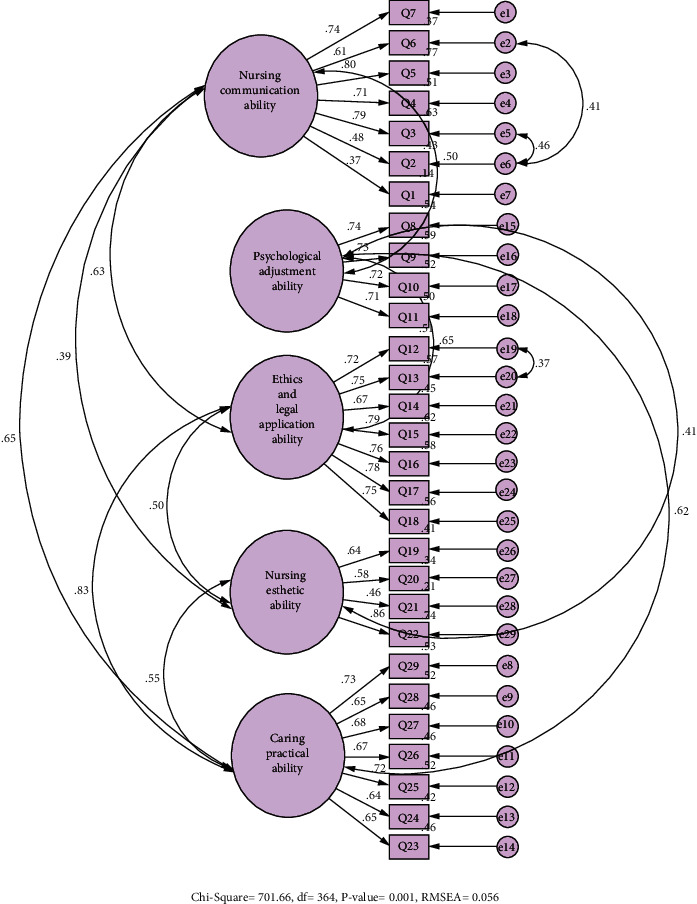
Path diagram of the factor structure of the scale.

*(3) Construct Validity*. Factor analysis, which is the most common method used to test construct validity, is a process to determine whether or not it is possible to group the items in a scale under different dimensions. Factor analysis has two types: exploratory factor analysis (EFA) and confirmatory factor analysis (CFA). EFA is performed to determine the number of subscales under which the items can be grouped. CFA is performed to test whether this structure is confirmed or not [24]. Before the construct validity analysis, the KMO test and Bartlett's test of sphericity were performed in order to determine whether or not the sample size was adequate. A KMO test value of <0.50 is considered to be unacceptable, a value between 0.80 and 0.90 is good, and a value of >0.90 is very good [25]. For factor analysis of the scale, Principal Component Analysis and Varimax rotation, which are the most common factor analysis statistical techniques, were used. After the factor analysis, the view that items should have factor loadings of at least 0.30 and it would be more appropriate to remove the items below these values was taken into account [26]. Following the exploratory factor analysis, the findings of the subscales in the scale were tested with confirmatory factor analysis (CFA). Values determined for X2/SD as <5, for the RMSEA as <0.08, and for the CFI and IFI as >0.90 after the CFA are considered as the lower limits of the data fit index of the model [27–29].  Figure 1 shows the CFA path diagram of the HPANS after the CFA model.

*(4) Reliability*. In a scale, internal consistency and homogeneity of the items are tested with Cronbach's *α* internal consistency coefficient technique. A higher Cronbach's *α* reliability coefficient signifies that the items in the scale are more consistent and test the elements of the same feature [30, 31]. In the literature, it is stated that Cronbach's *α* reliability of ≥0.70 indicates that this scale can be used in characteristic studies [31, 32]. The correlation between the scores obtained from the scale items and the total score of the test was examined with item-total correlation coefficients. The suggestion stating that the acceptable coefficient should be ≥ 0.30 was taken into consideration during the selection of the item.

*(5) Stability over Time (Test-Retest)*. Stability over time (test-retest) is that the measurement procedure yields consistent results on repeated trials. Correlation analysis is used to evaluate the results of the two tests. A correlation coefficient closer to 1 shows that the test has better stability over time [33]. In order to test its test-retest characteristics, the HPANS was administered to 30 individuals at an interval of two weeks. 30 nurses who underwent retesting were excluded from the study.

### 2.3. Data Analysis

SPSS 23.0 and AMOS v20 packaged software was used to analyze the data. Numbers, percentages, standard deviation, and minimum and maximum values were used to analyze demographic characteristics of the participants. In the validity study of the HPANS, expert opinions, Bartlett's test of sphericity, Kaiser–Meyer–Olkin test (KMO), exploratory factor analysis, Principal Component Analysis, and confirmatory factor analysis were used to determine the content and construct validity. In reliability study of the HPANS, internal consistency and homogeneity were determined with Cronbach's alpha (*α*) coefficient, Pearson's correlation analysis, and item-total score correlation.

### 2.4. Ethical Considerations

The study was carried out in line with the principles of the Declaration of Helsinki. Written permission was obtained from the author of the scale via e-mail. In order to adapt the HPANS into Turkish in the present study, approval from the Ethics Committee of Istanbul Sabahattin Zaim University was obtained (dated March 26, 2021: numbered 2021/03).

## 3. Results

### 3.1. Sample Characteristics

The mean age of the nurses participating in the study was 31.2 ± 8.3 years, and their mean duration of working in the profession was 9.1 ± 9.0 years. Of the participants, 80.7% were female, 55.3% were single, 68.7% had a Bachelor's degree, and 32.7% were working in the internal diseases service (Table 1).

### 3.2. Validity Analysis

In the present study, Bartlett's test of sphericity analysis was carried out and then the KMO test value was calculated as 0.92 and the X2 value was calculated as 4525.73. The results were significant at the significance level of *p* ≤ 0.001 (Table 2). Sample size was determined to be adequate and suitable for the factor analysis. Varimax rotation technique was used for factor analysis.

The analysis showed a 5-factor structure. According to the analysis results, items 23–29 were collected under the first factor which accounted for 14.28% of the total variance, items 1–7 were collected under the second factor which accounted for 13.15%, items 12–18 were collected under the third factor which accounted for 12.81%, items 8–11 were collected under the fourth factor which accounted for 10.76%, and items 19–22 were collected under the fifth factor which accounted for 8.76%. Thus, it was found that 59.63% of the total variance was explained by the five-factor scale (Table 3).

Confirmatory factor analysis (CFA) fit index values of the HPANS were determined as follows: *X*2 = 701.66, df = 364 (*p* ≤ 0.001), X2/df = 2.37, RMSEA = 0.056, CFI = 0.92, RMR = 0.036, SRMR = 0.054, NFI = 0.85, IFI = 0.92, and TLI = 0.91 (Table 4). Accordingly, the model fit of the scale was considered acceptable.

Following EFA and CFA, the Turkish version of five-subscale HPANS was confirmed without making any change. All the findings showed that the scale was highly valid for the Turkish culture.

### 3.3. Reliability Analysis

In the analysis performed to find out reliability, the data collection tools were reapplied to 30 participants from the sample for whom the EFA was performed 2 weeks later. 30 nurses who underwent retesting were excluded from the study. The pretest-posttest correlation coefficient was found as 0.815 for the overall scale, 0.990 for the nursing communication ability subscale, 0.852 for the psychological adjustment ability subscale, 0.941 for the ethics and legal application ability subscale, 0.730 for the nursing esthetic ability subscale, and 0.805 for the caring practical ability subscale (Table 5). These results show a high external reliability and a stable structure. [34] In addition, Cronbach's *α* internal consistency coefficient was tested to determine the internal reliability of the scale. It was 0.930 for the overall scale, 0.850 for the nursing communication ability subscale, 0.822 for the psychological adjustment ability subscale, 0.897 for the ethics and legal application ability subscale, 0.711 for the nursing esthetic ability subscale, and 0.858 for the caring practical ability subscale (Table 3). These values show high internal consistency level [25]. Item-total correlation coefficients were found to be above 0.30 (0.33–0.68).

## 4. Discussion

Our search for a specific scale used to measure humanistic practice ability demonstrated the lack of such a scale in Turkey. Therefore, the present study aimed to adapt the Humanistic Practice Ability of Nursing Scale (HPANS) developed by Zhang et al. [19] to Turkish and to perform its validity and reliability study for the Turkish population. In this section, the findings related to HPANS, which consist of 29 items and 5 subscales, were discussed.

### 4.1. Results on the Validity of the HPANS

Content validity was determined by taking the opinions of 5 experts and turning the results, obtained according to Davis technique, into quantitative data so that CVI was calculated [23]. According to the measurement results, the total CVI value was found as 0.96. Considering that the limit value of total CVI is 0.80 in content validity, this result shows that the CVI of each item in this scale is high and sufficient [35].

The Kaiser–Meyer–Olkin test (KMO) and Bartlett's test of sphericity were performed to evaluate whether or not the sample size was suitable for determining the construct validity of the HPANS. As is stated in the literature, a KMO test value of <0.50 is regarded unacceptable, a value between 0.80 and 0.90 is good, a value of >0.90 is very good, and the result of Bartlett's test of sphericity should be significant. In the present study, the KMO test value was 0.92 and the results of the Bartlett's test of sphericity (*X*2 = 4525.73; *p* ≤ 0.001) were significant [25]. In the original scale study by Zang and others [19], the KMO test value was calculated as 0.95 and the results of the Bartlett's test of sphericity were as follows: *X*2 = 35882.97 (*p* ≤ 0.001) [19]. These results showed that the data were sufficient for factor analysis. The HPANS's factor structure was found using the “Principal Component Analysis method” and “Varimax orthogonal rotation method.”

After the analysis, a 5-factor (subscale) structure with an eigenvalue above 1.00 and explaining 59.63% of the total variance of the 29-item HPANS emerged. In the original version of the HPANS, it was also found that the rate of the total variance explained was 78.2%, the eigenvalue was >1, and it consisted of 5 subscales. It was determined that as in the original version of the HPANS, its Turkish version consisted of 5 subscales and its factor structure was adequate. In this study, item factor loadings were between 0.47 and 0.79. In the original version of the scale [19], all items had factor loadings of ≥ 0.40. In the literature, it is stated that the factor loadings of the items should be at least 0.30 and it would be more appropriate to remove the items whose factor loadings are below this value [26]. In the present study, there were no items with a factor loading below 0.30, which is compatible with the literature; thus, no items were removed from the scale.

In the study by Zang and others [19], CFA fit index values were as follows X2/df = 2.99, RMSEA = 0.05, CFI = 0.92, AGFI = 0.89, NFI = 0.97, IFI = 0.92, CFI = 0.98, RFI = 0.97, and TLI = 0.98, and they stated that these values indicated a good fit. In the present study, CFA fit index values of the HPANS were as follows: *X*2 = 701.66, df = 364 (*p* ≤ 0.001), X2/df = 2.37, RMSEA = 0.056, CFI = 0.92, RMR = 0.036, SRMR = 0.054, IFI = 0.92, and TLI = 0.91 (Table 4). It is stated in the literature that values of RMSEA of <0.08, RMR and SRMR of <0.05, *χ*^2^/df of <5, and IFI, CFI, and TLI of >0.90 are acceptable for the model [27–29]. These values indicate that the model data fit was high and acceptable.

### 4.2. Results on the Reliability of the HPANS

It is stated in the literature that Cronbach's alpha value is between 0.00 and 1.00, and as the value gets closer to 1.00, reliability increases and values > 0.70 are good and acceptable [25, 36]. In Zang et al.' s study [19], Cronbach's alpha (*α*) coefficient was 0.96 for the HPANS and ranged between 0.87 and 0.98 for its subscales [19]. In the present study, Cronbach's *α* internal consistency coefficient was 0.930 for the overall scale, 0.850 for the nursing communication ability subscale, 0.822 for the psychological adjustment ability subscale, 0.897 for the ethics and legal application ability subscale, 0.711 for the nursing esthetic ability subscale, and 0.858 for the caring practical ability subscale (Table 3). These values showed that the scale had a high level of internal consistency.

The results of the analysis demonstrated that all of the item-total score correlation coefficients were significant at *p* ≤ 0.001 and the item-total correlation coefficient values of the items ranged between 0.33 and 0.68. In the literature, it is stated that the acceptable value for the item-total correlation coefficient should be ≥ 0.30 [30]. These results showed that there was no problem in any of the scale items and the scale was reliable.

In the analysis to find out reliability, the data collection tools were reapplied to 30 participants from the sample for whom the EFA was performed 2 weeks later. In the literature, test-retest correlation coefficient values are classified as follows: 0.0–0.2, weak; 0.21–0.40, mild; 0.41–0.60, moderate; 0.61–0.80, sufficient; and 0.81–1, almost perfect [34, 37].

In the present study, the pretest-posttest correlation coefficient was found as 0.815 (*p* ≤ 0.001) for the overall scale. It was 0.990 (*p* ≤ 0.001) for the nursing communication ability subscale, 0.852 (*p* ≤ 0.001) for the psychological adjustment ability subscale, 0.941 (*p* ≤ 0.001) for the ethics and legal application ability subscale, 0.730 (*p* ≤ 0.001) for the nursing esthetic ability subscale, and 0.805 (*p* ≤ 0.001) for the caring practical ability subscale (Table 5). In the study, when the fit between the test-retest mean scores of the scale was examined, a positive and highly significant correlation was found. This result showed that the scale was not affected by time and measured the same situation even if time passed. The scale has high external reliability level and a stable structure.

## 5. Conclusion

The Turkish version of the HPANS with 29 items and 5 subscales was in good EFA and CFA agreement with the original version of the scale. The 5-factor structure of the scale was confirmed with results. Cronbach's *α* internal consistency coefficient, item-total correlation, and test-retest analysis of the scale were considered adequate. The results indicate that the HPANS is a valid and reliable tool to measure nurses' humanistic practice ability. It is thought that the HPANS would fill the gap in this area and would facilitate the decision to provide the necessary training when the need arises.

## Figures and Tables

**Table 1:** Demographic characteristics of the nurses (*n* = 300).

Variables	*N*	%
*Sex*		
Female	242	80.7
Male	58	19.3
*Marital status*		
Married	134	44.7
Single	166	55.3
*Educational level*		
High school	19	6.3
Associate degree	31	10.3
Bachelor's degree	206	68.7
Postgraduate	44	14.7
*Unit they worked in*		
Internal diseases service	98	32.7
Surgical service	38	12.7
Emergency room	47	15.7
Intensive care unit	33	11.0
Operating room	23	7.7
Community health center	24	8.0
Obstetrics service	8	2.7
Outpatient clinics	10	3.3
Administrative units	10	3.3
Pediatric service	9	3.0
	*X̄* ± SD	
Age (years)	31.2 ± 8.3 (min = 15.0 − max = 56.0)	
Duration of working in the profession (years)	9.1 ± 9.0 (min = 1.0 − max = 38.0)	

**Table 2:** Results of the Kaiser–Meyer–Olkin measure of sampling adequacy and Bartlett's test of sphericity.

Test	Results	
Kaiser–Meyer–Olkin measure of sampling adequacy	0.92	*p* ≤ 0.001
Bartlett's test of sphericity approx. Chi-square	4525.73	
Df	406	
Sig.	≤0.001	

**Table 3:** Mean score, item-total score correlation coefficients, factor loadings, alpha coefficients, and explained variance of the HPANS.

Items	Factor 1	Factor 2	Factor 3	Factor 4	Factor 5	Mean (SD)	Corrected item-total correlations	Cronbach's *α* if item deleted
Q23	0.688					4.50 (0.66)	0.579	0.928
Q24	0.684					4.38 (0.75)	0.579	0.929
Q27	0.679					4.31 (0.79)	0.576	0.929
Q25	0.631					4.16 (0.72)	0.552	0.928
Q29	0.620					4.11 (0.73)	0.645	0.928
Q28	0.587					3.88 (0.89)	0.645	0.928
Q26	0.555					3.96 (0.77)	0.553	0.928
Q3		0.797				3.99 (0.88)	0.627	0.928
Q5		0.746				4.01 (0.81)	0.660	0.927
Q2		0.720				3.87 (1.01)	0.460	0.930
Q4		0.658				4.01 (0.87)	0.533	0.929
Q7		0.649				4.08 (0.81)	0.568	0.928
Q6		0.580				3.83 (0.94)	0.603	0.928
Q1		0.471				4.10 (0.91)	0.335	0.932
Q17			0.732			4.26 (0.72)	0.607	0.928
Q15			0.713			4.41 (0.66)	0.652	0.928
Q12			0.682			4.35 (0.70)	0.667	0.927
Q13			0.651			4.21 (0.73)	0.689	0.927
Q14			0.573			4.65 (0.57)	0.574	0.929
Q16			0.549			4.25 (0.73)	0.681	0.927
Q18			0.542			4.05 (0.78)	0.677	0.927
Q10				0.778		3.90 (0.83)	0.496	0.929
Q11				0.756		3.73 (0.86)	0.503	0.929
Q9				0.725		3.80 (0.80)	0.559	0.929
Q8				0.675		3.84 (0.76)	0.590	0.928
Q22					0.783	3.75 (1.03)	0.508	0.929
Q20					0.740	3.84 (1.14)	0.329	0.933
Q19					0.718	3.21 (1.13)	0.456	0.930
Q21					0.488	3.84 (1.14)	0.371	0.932
Eigenvalue explained	4.11	3.81	3.71	3.10	2.54			
Variance (%) total	14.20	13.15	12.81	10.76	8.76			
Variance (%)	59.63							
Cronbach's *α*	0.850	0.822	0.897	0.711	0.858	Total = 0.930		

**Table 4:** Results of the confirmatory factor analysis.

Fit criteria	Found	Appropriate	Acceptable
*X*2/df	1.928	<2	<5
RMSEA	0.056	<0.05	<0.08
CFI	0.92	>0.95	>0.90
TLI	0.91	>0.95	>0.90
IFI	0.92	>0.95	>0.90
RMR	0.036	<0.05	<0.08
SRMR	0.054	<0.05	<0.08

CFI: Comparative Fit Index; RMSEA: Root Mean Square Error of Approximation; RMR: Root Mean Square Residual; NFI: Normed Fit Index; IFI: Incremental Fit Index; TLI: Tucker–Lewis Index; SRMR: Standardized Root Mean Square Residual.

**Table 5:** Results of test-retest reliability.

	*X̄* ± SD		*t*	*p*	*r*	*p*
*Factor* 1	Pretest	28.30 ± 4.17	0.238	0.813	0.990	≤0.001
	Posttest	28.23 ± 4.04				

*Factor* 2	Pretest	15.33 ± 2.66	−0.367	0.717	0.852	≤0.001
	Posttest	15.43 ± 2.80				

*Factor* 3	Pretest	30.86 ± 3.77	−0.283	0.779	0.941	≤0.001
	Posttest	30.66 ± 3.76				

*Factor* 4	Pretest	15.50 ± 2.71	−0.280	0.781	0.730	≤0.001
	Posttest	15.60 ± 2.59				

*Factor* 5	Pretest	29.53 ± 4.56	0.454	0.653	0.805	≤0.001
	Posttest	29.30 ± 4.42				

*Total*	Pretest	119.53 ± 14.87	0.238	0.813	0.815	≤0.001
	Posttest	119.13 ± 15.31				

*t*: dependent-samples *t*-test, *r*: Pearson's correlation coefficient, and *p*: significance level.

## Data Availability

Data share is not applicable to this manuscript.
